# Combined branch retinal artery and branch retinal vein occlusion from a globe penetrating nail gun injury

**DOI:** 10.3205/oc000113

**Published:** 2019-06-18

**Authors:** Bryon R. McKay, James A. Martin

**Affiliations:** 1 Department of Ophthalmology and Vision Sciences, The University of Toronto, Toronto, Ontario, Canada; 2 Department of Eye Medicine & Surgery, McMaster University & St. Joseph’s Healthcare, Hamilton, Ontario, Canada

**Keywords:** branch retinal artery occlusion, branch retinal vein occlusion, nail injury, open globe injury, retinal detachment, trauma

## Abstract

**Background:** Nail gun injuries represent a significant proportion of work-related ocular trauma. Here we report a rare case of a combined branch retinal arterial occlusion (BRAO) and branch retinal venous occlusion (BRVO) from a nail gun injury in a patient who was wearing eye protection.

**Case description:** A 23-year-old male presented with a left globe penetrating injury from a pneumatic nail gun. The nail ricochet bypassed the patient’s protective eyewear, penetrating the left globe causing multiple retinal tears and a combined BRAO and BRVO in the inferior temporal vascular arcade. The patient underwent prompt surgical repair obtaining an excellent anatomic outcome. However, the visual outcome was 20/200 OS at one and two years post injury primarily due to extensive ischemic damage of the retina.

**Conclusion:** This case is the first to describe a combined BRAO and BRVO from a globe penetrating injury. What makes this case even rarer is that the patient was wearing eye protection at the time of the injury. Despite appropriate emergency management, rapid referral for ophthalmologist assessment, and timely surgical management of this patient, the visual outcome was poor. The vascular injury ultimately compromised a significant segment of the retina, including the macula.

## Background

Ocular trauma from globe penetrating injury and intraocular foreign bodies (IOFBs) constitute approximately 17–44% of cases of significant vision impairing traumatic ocular injury [1], [2]. Ocular trauma is a major cause of monocular blindness worldwide and the second most common cause of visual impairment in the United States [1], [3], with nail-related eye injury accounting for approximately 5% of major eye injuries [4].

The use of pneumatic nail guns is common place in carpentry and construction. Despite strict safety guidelines with the correct operation requiring the use of safety glasses, the rates of non-compliance with safety glasses in the literature range from 56% to 94% [5], [6], [7], [8], [9]. There are few reports of significant ocular injury in patients wearing correct personal protective equipment (PPE). In a recent study looking at 603 eye injuries, only 3 patients were wearing PPE at the time of the injury [10].

Here we present an unusual case of a man who sustained a work-related globe penetrating injury from a nail fired from a pneumatic nail gun causing a combined branch retinal arterial and branch retinal vein occlusion (BRAO and BRVO). Interestingly, the patient was wearing eye protection and this injury was sustained by a nail ricochet that bypassed the patient’s safety glasses, penetrating the globe inferionasally.

## Case description

**Figure 1:**
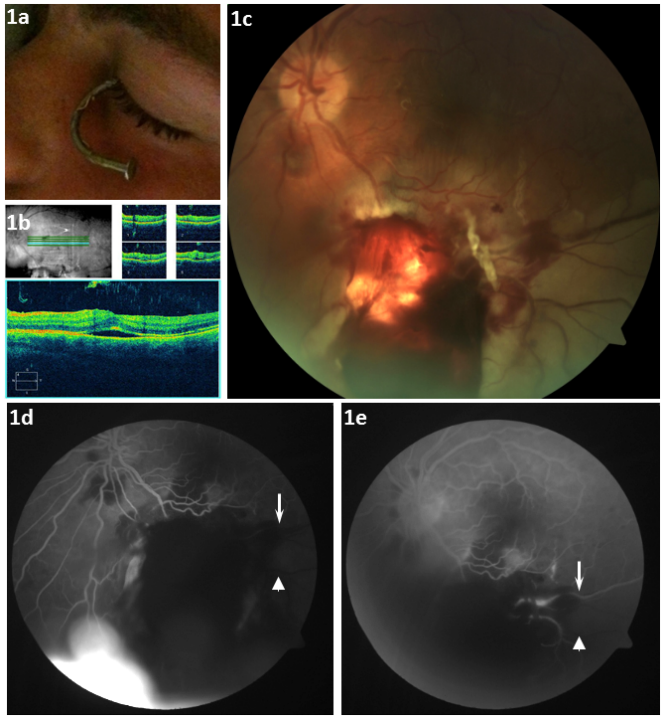
Initial presentation and subsequent fundus photo and OCT 3 d post injury. a) Left eye (OS) with nail *in situ* (bent from ricochet). b) OCT analysis (OS) 3 d post-injury: subretinal fluid. c) Fundus photo: large inferior central retina tear, vitreous hemorrhage and combined BRAO and BRVO 3 d post-injury (note pallor of the inferior retina). d) Fluorescein angiography: arterial-venous phase 3 d post-injury illustrating a lack of arterial perfusion (arrowhead: arteriole; arrow: venule). e) Retrograde venous filling in the late venous phase (arrowhead: arteriole shows no filling; arrow: venule shows retrograde flow)

A 23-year-old male presented to the emergency department following a work-related globe penetrating injury from a pneumatic nail gun to his left eye (OS) 4 mm inferionasally from the limbus (Figure 1a (Fig. 1)). At the time of the injury, the patient was operating a pneumatic nail gun in an overhead position at a housing contraction site in Ontario Canada. The patient was wearing standard eye protective glasses (polycarbonate lenses, CSA approved) at the time of the nail ricochet. He was promptly referred to Ophthalmology for emergency nail removal and primary closure and subsequently underwent retinal specialist review 3 days later. 

### On examination

Best corrected visual acuity (BCVA) was 20/20 OD and 20/70 OS. Pupils were 5 mm OD and 6 mm OS. The left pupil was nonreactive to direct light; with no relative afferent papillary defect. Extraocular movements and intraocular pressures were normal. Slit-lamp examination demonstrated a traumatic cataract, and 1+ flare in the anterior chamber. Fundus examination demonstrated a posterior vitreous hemorrhage, 4 large semi-circular retinal holes at the 4:00, 5:00, 8:00 and 9:00 meridians between the ora serrata and the equator, each with a sub-retinal fluid (SFR) cuff as well as a large retinal hole (4 disc diameters) inferior to the optic nerve along the 6:00 meridian, two horseshoe retinal tears along the 12:00 meridian and extensive commotio in the inferior and inferotemporal retina (Figure 1b,c (Fig. 1)). Optical coherence tomography (OCT) demonstrated SRF in the macular region (Figure 1b (Fig. 1)) and fluorescein angiography demonstrated a BRAO and BRVO along the inferotemporal vascular arcade (Figure 1d,e (Fig. 1)) with no branch arterial filling and delayed branch venous filling by retrograde flow.

### Operative repair 

11 days after the injury the patient underwent 23G vitrectomy combined with cataract extraction with IOL, scleral buckle, silicone oil tamponade and laser endophotocoagulation without complication. Five days post-surgery BCVA was 20/100 OS with no SRF on examination and OCT. Silicone oil was removed 3 months later and replaced with SF6 gas without complication. 

### Visual outcome

**Figure 2:**
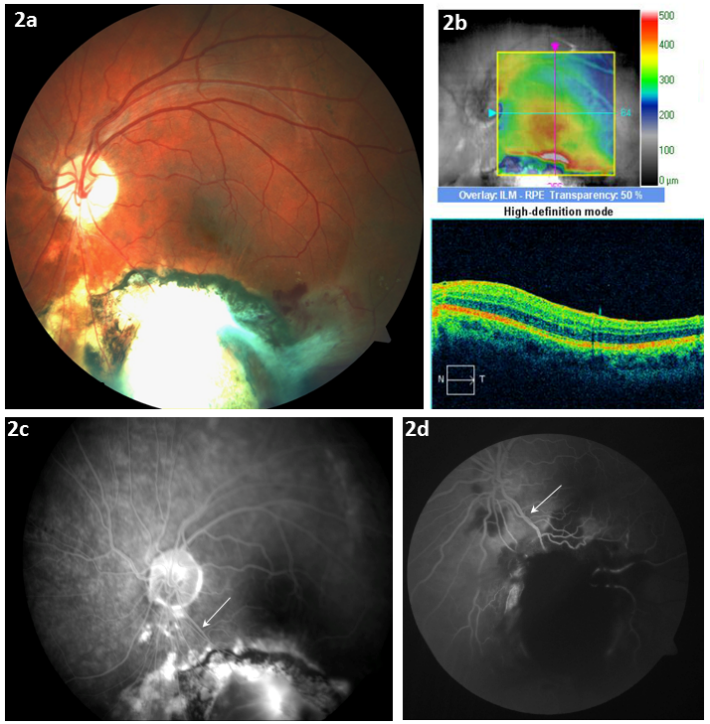
One-year follow-up demonstrates marked scaring of the inferior central retina. a) Fundus photo (OS) 1 y post-injury: marked fibrosis of the inferior retina extending into the inferior macula with marked attenuation of the inferior temporal vascular arcade. b) Corresponding OCT: resolution of sub-retinal fluid and thickening of the inferior peri-macular area. c and d) Angiography 1 y post-injury: (c) extensive vascular attenuation in the inferior circulation compared to the time of the injury (d) – (arrow denotes same vessel).

Over the next 4 months, the patient complained of increasing blurred vision OS which decreased from best corrected visual acuity of 20/100 OS to 20/400 OS. Increased capsular haze was noted and treated with a YAG laser capsulotomy. Despite a good surgical outcome, BCVA 1 year after injury was 20/400 OS with scaring and atrophy evident in the inferior retina (Figure 2a,b (Fig. 2)). Extensive attenuation of the inferior vascular arcade was noted compared to the time of injury (Figure 2b,c (Fig. 2)). The extent of tissue ischemia from the vascular injury is likely responsible for the patient’s poor visual outcome.

## Discussion

Eye injury from nails accounts for approximately 5% of all major eye injuries [4], with a large proportion of these injuries being associated with nail guns. In the majority of cases, ocular nail gun injuries are associated with the lack of proper personal protection equipment (PPE) or the improper use of such equipment [3]. Interestingly, our patient had been wearing eye protection at the time of injury with the mechanism most likely due to the nail ricocheting at an angle that bypassed his PPE. Nail ricochet is common and represent 37% of nail gun related eye injuries [3]. Nail guns can propel nails with a velocity of 100–150 m/s [11] causing them to act as missiles. Typically, injuries caused by nail guns produce less contusive damage to the retina versus blunt force-type injuries and therefore are often less severe than expected [12]. Ricochet injuries are less predictable because the nail is commonly bent [11] and the trajectory can be complicated by rotational forces. Although studies suggest favourable visual acuity is achieved in the majority of nail gun injury cases [3], patients with posterior segment injury tend to have worse visual outcomes [3]. In our patient, there was extensive retinal detachment with a large tear encroaching on the periphery of the inferior macular region. However, even with the direct retinal trauma the majority of the macula and fovea appeared to be spared from direct trauma. Unfortunately for our patient, the nail also disrupted the arterial and venous supply to the inferior portion of the retina leading to secondary ischemic damage.

## Conclusions

This case is unusual because reports of traumatic combined BRAO and BRVO are rare, with only one other report in the literature [13] and there are no reports of such an injury occurring with the use of PPE. What is rarer still is that the nail did not break the safety-lens but rather the angle of ricochet allowed the nail to enter the globe from below the safety-glasses, unimpeded by the lens. This injury reinforces the importance of workplace education on the proper usage of nail guns as well as the proper use of PPE and demonstrates that despite prompt and appropriate medical and surgical treatment, globe penetrating trauma can have catastrophic sequelae.

## Abbreviations

- BCVA – best corrected visual acuity
- BRAO – branch retinal artery occlusion
- BRVO – branch retinal vein occlusion
- IOFB – intraocular foreign body
- IOL – intraocular lens
- OCT – optical coherence tomography
- OD – right eye
- OS – left eye
- PPE – personal protective equipment
- SFR – subretinal fluid

## Notes

### Patient consent

The patient has viewed the content and images of this case report and has consented to the submission of the case report for publication.

### Competing interests

The authors certify that they have NO affiliations with or involvement in any organization or entity with any financial interest (such as honoraria; educational grants; participation in speakers’ bureaus; membership, employment, consultancies, stock ownership, or other equity interest; and expert testimony or patent-licensing arrangements), or non-financial interest (such as personal or professional relationships, affiliations, knowledge or beliefs) in the subject matter or materials discussed in this manuscript. Dr. Martin reports personal fees from Alcon Canada, personal fees from Bayer Canada, unrelated to the submitted work. Dr. McKay has nothing to disclose.

### Author contributions

JAM: Consultant retinal surgeon who conducted the referral, initial workup, surgical repair and further surgical management. BRM: Reviewed the case, prepared the manuscript and figures. Both JAM and BRM obtained the patients consent for publishing and revised the manuscript and gave final approval for publication.
